# Neural Mechanism of 5-HT4R-Mediated Memory Enhancement in Hippocampal–Prefrontal Circuits in a Mouse Model of Schizophrenia

**DOI:** 10.3390/ijms26083659

**Published:** 2025-04-12

**Authors:** Thomas Gener, Sara Hidalgo-Nieves, Cristina López-Cabezón, Maria Victoria Puig

**Affiliations:** 1 Department of Neuroscience and Experimental Therapeutics, Institute of Biomedical Research of Barcelona, CSIC, 08036 Barcelona, Spain; thomas.gener@iibb.csic.es (T.G.); sara.hidalgo@iibb.csic.es (S.H.-N.); cristina.lopez@iibb.csic.es (C.L.-C.); 2 Catalan Institute of Nanoscience and Nanotechnology (ICN2), CSIC and BIST, Campus UAB, 08193 Bellaterra, Spain

**Keywords:** hippocampus, prefrontal cortex, neural oscillation and synchrony, theta and gamma oscillations, phase coherence, novel object recognition test, short-term and long-term memory

## Abstract

We investigated the cellular and neurophysiological mechanisms underlying the pro-cognitive effects of 5-HT4R activation in hippocampal–prefrontal pathways. Our findings show that, in addition to pyramidal neurons, 30–60% of parvalbumin+ interneurons in the CA1, CA3, and dentate gyrus (DG) of the hippocampus and the anterior cingulate (ACC), prelimbic (PL), and infralimbic (IL) regions of the prefrontal cortex co-express 5-HT4Rs. Additionally, 15% of somatostatin+ interneurons in CA1 and CA3 express 5-HT4Rs. Partial 5-HT4R agonist RS-67333 (1 mg/kg, i.p.) exerted anxiolytic effects and ameliorated short-term (3-min) and long-term (24-h) memory deficits in a mouse model of schizophrenia-like cognitive impairment induced by sub-chronic phencyclidine (sPCP) but did not enhance memory in healthy mice. At the neurophysiological level, RS-67333 normalized sPCP-induced disruptions in hippocampal–prefrontal neural dynamics while having no effect in healthy animals. Specifically, sPCP increased delta oscillations in CA1 and PL, leading to aberrant delta–high-frequency coupling in CA1 and delta–high-gamma coupling in PL. RS-67333 administration attenuated this abnormal delta synchronization without altering phase coherence or signal directionality within the circuit. Collectively, these results highlight the therapeutic potential of 5-HT4R activation in pyramidal, parvalbumin+, and somatostatin+ neurons of hippocampal–prefrontal pathways for mitigation of cognitive and negative symptoms associated with schizophrenia.

## 1. Introduction

Serotonin (5-hydroxytryptamine, 5-HT) is a key neuromodulator involved in the regulation of mood, cognition, and various physiological functions. Among its receptor subtypes, the 5-HT4 receptor (5-HT4R) plays crucial roles in mood regulation, memory, and food intake [1,2,3,4,5]. Due to its distinct cerebral distribution and physiological properties, 5-HT4R has emerged as a promising target for brain disorders such as depression, Alzheimer’s disease, eating disorders, and Parkinson’s disease [2,5,6,7,8]. Preclinical studies show that 5-HT4R activation produces rapid antidepressant-like effects and enhances cognitive performance in rodent models [1,5,6,9,10,11,12], yet the underlying neural mechanisms remain unclear.

5-HT4Rs are coupled to G proteins that stimulate cAMP production, promoting neuronal excitability. They rapidly modulate synaptic transmission and facilitate synaptic plasticity, including learning-induced dendritic spine growth in the hippocampus (HPC) [13,14]—processes that are essential for learning and memory. In both humans and rodents, 5-HT4Rs are predominantly expressed in the basal ganglia, with lower levels in the hippocampus and frontal cortex, where they localize to pyramidal neurons, interneurons, and presynaptic terminals [15]. In the human frontal cortex, receptor binding exhibits a distinct laminar pattern, with higher expression in superficial layers [16,17,18,19,20].

We investigated the cellular mechanisms underlying the pro-cognitive effects of 5-HT4R activation within hippocampal–prefrontal pathways, a key circuit for learning and memory. Both regions receive dense serotonergic innervation from the median and dorsal raphe nuclei, respectively, and express multiple serotonin receptor subtypes, including 5-HT4Rs [21,22,23]. In mouse models of psychiatric and neurodevelopmental disorders, hippocampal–prefrontal neural dynamics are disrupted alongside memory deficits that can be restored by pharmacological and non-pharmacological memory-enhancing interventions [24,25,26]. Notably, hippocampal–prefrontal circuits exhibit intrinsic synchronization within the theta (8–12 Hz) and gamma (30–100 Hz) frequency ranges, which are critical for cognitive function. Our recent findings demonstrate that the directionality of information flow at these frequencies strongly correlates with memory performance (R = 0.7 between high-gamma mPFC → dHPC signaling and short-term memory performance and R = −0.72 between theta dHPC → mPFC signaling and long-term memory performance) and are modulated by serotonin receptors 5-HT1A and 5-HT2A, as well as by atypical antipsychotic drugs targeting these receptors [27,28].

In this study, we examined the distribution of 5-HT4Rs across key hippocampal regions (CA1, CA3, and DG) and medial prefrontal cortex (mPFC) subregions (cingulate, prelimbic, and infralimbic cortices). We also quantified their co-expression in parvalbumin (PV) and somatostatin (SST) interneurons, two inhibitory neuron subtypes essential for theta and gamma synchronization [29,30,31]. Additionally, we investigated how the 5-HT4R agonist RS-67333—recognized for its memory-enhancing properties [1,5,9,10,12]—modulates circuit dynamics between the CA1 region of the dorsal hippocampus (dHPC) and the prelimbic (PL) mPFC in both healthy mice and a schizophrenia-related cognitive impairment model induced by sub-chronic phencyclidine (PCP) treatment [32,33]. By integrating neurophysiological and anatomical data from mice with intact and impaired memory function, this study provides novel insights into the role of 5-HT4Rs in mood and cognition, offering a foundation for future therapeutic strategies targeting these receptors in brain disorders.

## 2. Results

### 2.1. 5-HT4Rs Are Expressed by 30 to 60% of PV+ Interneurons in Mouse dHPC and mPFC and 15% of SST+ Neurons in the dHPC

We first aimed to gain insight into the expression pattern of 5-HT4Rs in the mouse dHPC and mPFC, as the available literature on this topic is incomplete compared to that of human and rat expression [34]. While human and rat studies have reported strong 5-HT4R expression in regions such as the basal ganglia, olfactory tubercle, and hippocampal formation, they indicated lower levels in the frontal cortex, with expression primarily localized to pyramidal neurons [16,23,34]. Given the low expression levels of 5-HT4Rs in the mouse dHPC and mPFC, we optimized our immunohistochemical protocols to achieve high signal-to-noise ratios of 5-HT4R detection, both alone and in combination with parvalbumin (PV) or somatostatin (SST) markers. For example, we incubated the primary antibodies over the weekend and employed advanced confocal microscopy techniques to capture the staining (for detailed methodology, see the Section 4). This approach allowed us to examine 5-HT4R expression not only in the soma but also on individual fibers with high signal-to-noise ratios, revealing the presence of 5-HT4Rs on both the somatic and dendritic surfaces of neurons.

**Figure 1:**
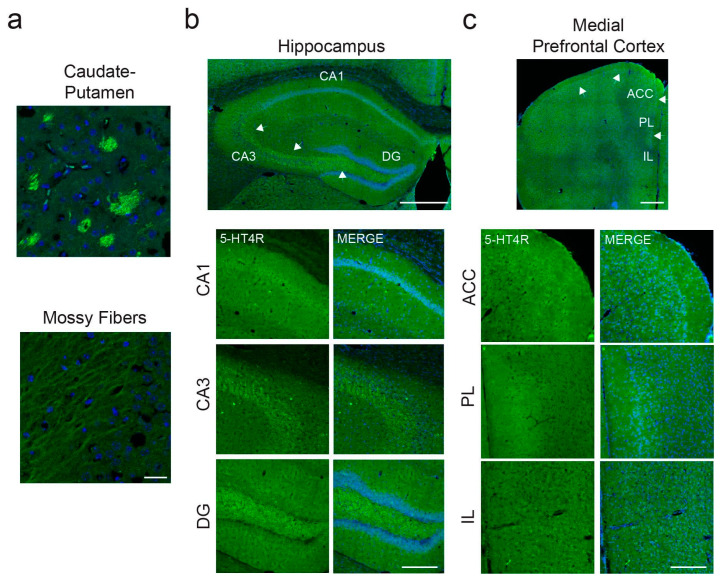
Strong expression of 5-HT4Rs in caudate–putamen and mossy fibers and lower levels in dHPC and mPFC. (**a**) High levels of 5-HT4Rs were detected in patches within the caudate–putamen and in individual mossy fibers within the hippocampal formation. Images were taken at 63×. Scale bar = 25 µm. (**b**) In the dHPC, the strongest expression was found in the pyramidal cell layer of CA1. In CA3 and DG, the most evident staining pertained to mossy fibers (white arrows), whereas pyramidal cells of the granule layer expressed very low levels of 5-HT4Rs. Top: Mosaic images taken at 20x. Scale bar = 500 µm. Bottom: Images taken at 20×. Scale bar = 200 µm. (**c**) In the mPFC, a clear 5-HT4R-expressing layer (2–3) could be detected in the ACC and PL cortices (white arrows). Expression in the IL cortex was very low. Top: Mosaic images taken at 20×. Scale bar = 500 µm. Bottom: Images taken at 20×. Scale bar = 200 µm.

Our findings confirmed strong 5-HT4R expression in the caudate–putamen and mossy fibers within the hippocampal formation (Figure 1a,b), as reported in the literature. In the dHPC, we observed moderate 5-HT4R expression in the pyramidal layer of CA1 and lower expression in CA3 and the dentate gyrus (DG; Figure 1b). In the mPFC, we detected low to moderate expression in the anterior cingulate cortex (ACC) and prelimbic cortex (PL), particularly in layers 2/3, while expression in the infralimbic cortex was scattered (IL; Figure 1c). This layer-specific expression pattern is consistent with findings in the human frontal cortex.

**Figure 2:**
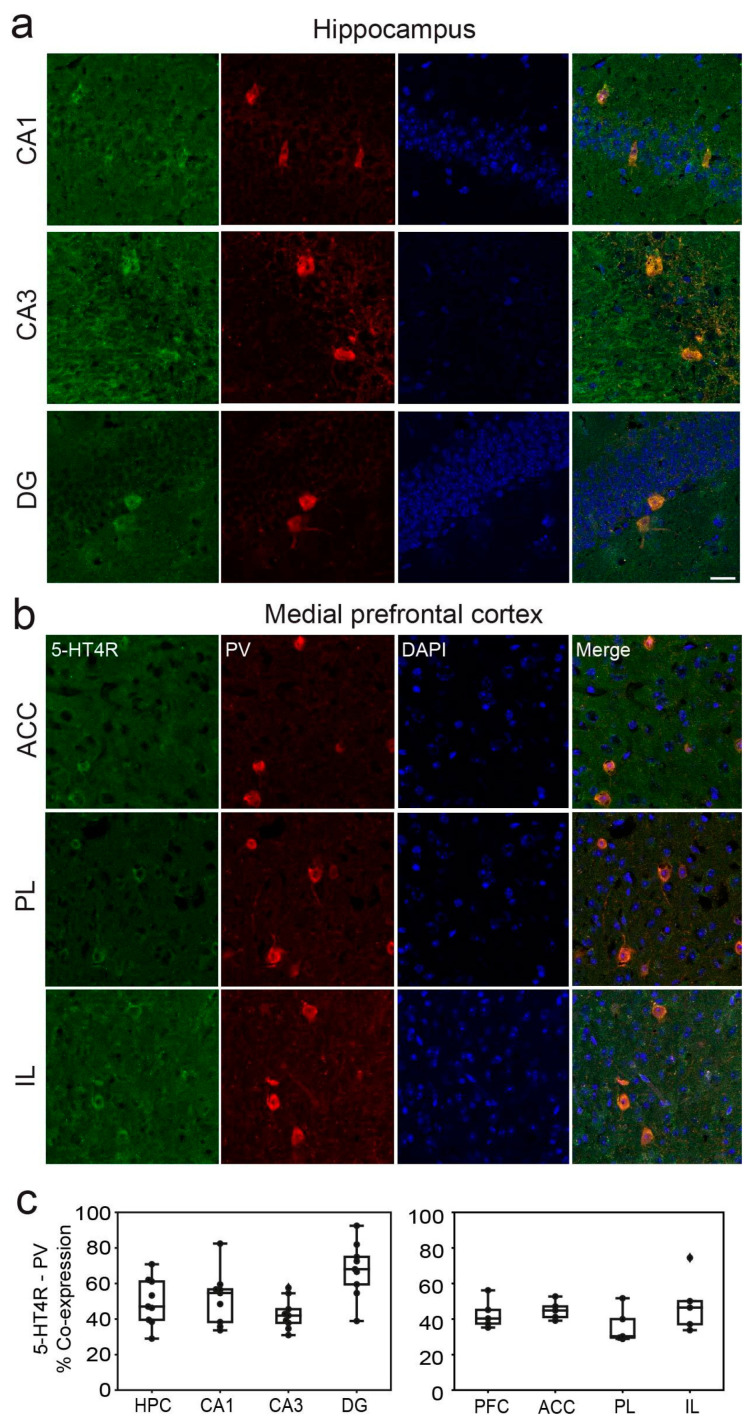
5-HT4R immunoreactivity in PV+ interneurons of the dHPC and mPFC. Representative images of PV cells (red) expressing 5-HT4R (green) and DAPI counterstaining (blue) in the distinct subregions of the dHPC (**a**) and the mPFC (**b**). Note the mossy fiber staining in CA3 and low 5-HT4R immunofluorescence in the mPFC. Images were obtained at 63x. Scale bar = 25 µm. (**c**) Quantification of 5-HT4R and PV immunofluorescence co-stainings in the distinct subregions of the dHPC and mPFC. See Table 1 for complementary information.

**Figure 3:**
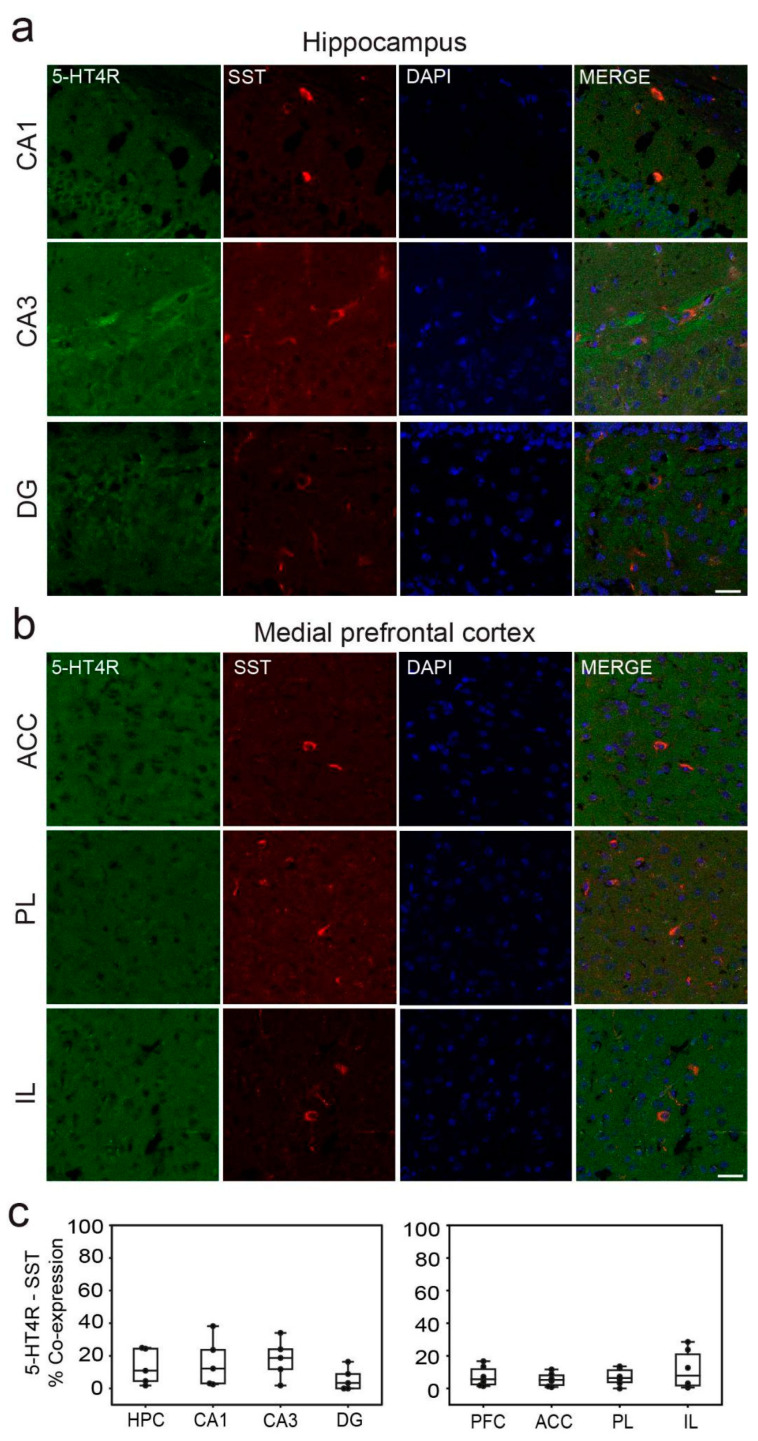
5-HT4R immunoreactivity in SST+ interneurons of the dHPC and mPFC. (**a**) Representative images of SST cells (red) expressing 5-HT4R (green) and DAPI counterstaining (blue) in the distinct subregions of the HPC. (**b**) Representative examples of SST cells negative for 5-HT4Rs. Co-expression of SST and 5-HT4Rs in the mPFC was rare. Images were obtained at 63x. Scale bar = 25 µm. (**c**) Quantification of 5-HT4R and SST immunofluorescence co-stainings in the distinct subregions of the HPC and mPFC. See Table 1 for complementary information.

In the dHPC, 5-HT4Rs were detected in 51.76 ± 6%, 42.79 ± 5%, and 67.74 ± 9% of parvalbumin-positive (PV+) cells in the CA1, CA3, and DG regions, respectively (Figure 2a). Additionally, a smaller fraction of cells co-expressed 5-HT4Rs and somatostatin (SST): 15.95 ± 7%, 18.11 ± 9%, and 5.71 ± 5% in the CA1, CA3, and DG regions, respectively (Figure 3a). It is worth noting that the number of PV+ and SST+ cells in the DG was smaller than in the other regions (Table 1). In the mPFC, co-expression of 5-HT4Rs and PV was found in 43.13 ± 6%, 32.31 ± 6%, and 42.82 ± 8% of cells in the ACC, PL, and IL regions, respectively (Figure 2b). Co-expression of 5-HT4Rs and SST was rare, detected in 5.51 ± 3%, 7.03 ± 4%, and 11.67 ± 6% of the ACC, PL, and IL regions, respectively (Figure 3b). There were no significant differences between female and male mice (Table 1; see original images in Supplementary Materials).

In summary, our findings reveal that approximately 30 to 60% of PV+ interneurons express 5-HT4Rs in both the dHPC and mPFC; 15% of expression was detected in SST+ interneurons of the dHPC, while expression in the mPFC was rare.

### 2.2. 5-HT4R Partial Agonist RS-67333 Improves Memory Performance and Reduces Anxiety in the sPCP Model of Schizophrenia

**Figure 4:**
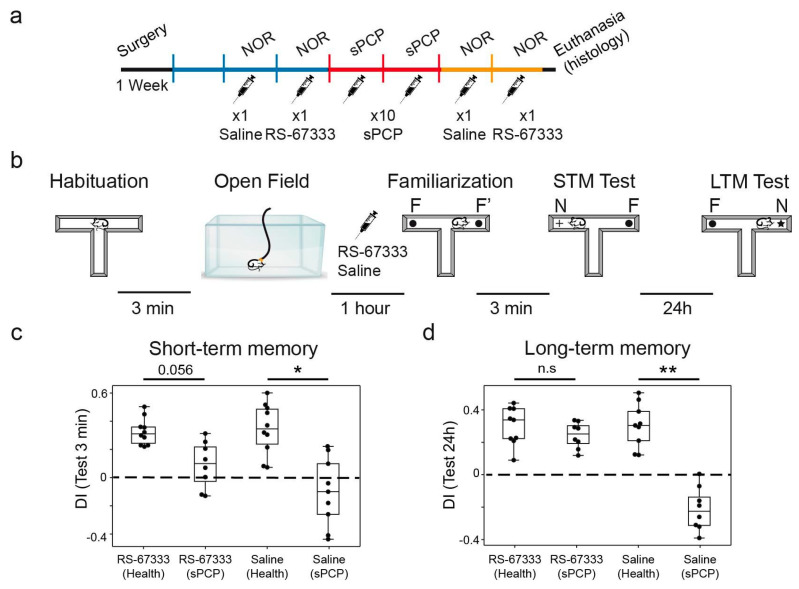
5-HT4R activation reduced sPCP-induced memory impairments in the sPCP model of schizophrenia. (**a**) Experimental protocol. Mice were implanted with recording electrodes in the CA1 and PL. During the first post-recovery week, mice were handled and became familiar with the recording cable. Saline and RS-67333 (1 mg/kg, i.p.) were injected before the familiarization phase of the NOR task for two consecutive weeks before and after subchronic treatment with PCP (10 mg/kg, s.c., 5 + 5 injections). (**b**) The four phases of the NOR task. Electrophysiological recordings were performed for 1 h prior to the familiarization phase. RS-67333 and saline were injected after a 15 min baseline, 45 min before familiarization. (**c**) RS-67333 improved STM but did not fully restore it to the level of healthy controls. Repeated-measures ANOVA followed by Bonferroni’s post hoc test (RS-67333, health vs. sPCP, *p* = 0.056; saline, health vs. sPCP, * *p* = 0.047). (**d**) RS-67333 fully restored LTM in sPCP-treated animals (RS-67333, health vs. sPCP, *p* = 1; saline, health vs. sPCP, ** *p* = 0.005). Data are presented as mean ± S.E.M.

We first validated the memory-enhancing and memory-rescuing effects of RS-67333. We hypothesized that acute administration of the 5-HT4R agonist before the familiarization phase of the NOR task would strengthen the acquisition of new memories, as previously reported for other memory-enhancing compounds [33,35]. We selected a dose of 1 mg/kg based on previous studies demonstrating the efficacy of this dose in enhancing place and object recognition memory in young adult rats [12]. We assessed short- and long-term recognition memory (STM and LTM, respectively) using the novel object recognition test (NOR), which capitalizes on mice’s natural preference for novel objects over familiar ones. This behavior is strongly dependent on the HPC, with recent studies, including our own, implicating hippocampal–prefrontal circuits in its encoding [24,28,36]. Nine mice were implanted with recording electrodes in the CA1 region of the dHPC and the PL mPFC using previously established methods [24,26,27]. After post-surgical recovery, animals were habituated to the recording cable while freely exploring an open field. STM and LTM were evaluated in both healthy conditions and after subchronic administration of NMDAR antagonist phencyclidine (sPCP; 10 injections at 10 mg/kg, s.c.; Figure 4a), a widely used model for schizophrenia-related cognitive deficits. The animals received either saline (controls) or RS-67333 (1 mg/kg, i.p.) 45 min prior to the familiarization phase in each NOR test, both before and after sPCP treatment (Figure 4b).

As anticipated, mice displayed positive discrimination indices (DIs; [time visiting the novel object—time visiting the familiar object]/total exploration time) for novel versus familiar objects following saline administration, confirming intact recognition memory under healthy conditions in both the 3 min (STM) and 24 h (LTM) intervals. A repeated-measures ANOVA assessing the effects of saline and RS-67333 before and after sPCP (four groups) revealed significant reductions in DIs following saline injections in sPCP-treated mice during the STM test, with RS-67333 partially ameliorating this impairment (*n* = 8 mice; F_1,7_ = 11.201, *p* = 0.002) (Figure 4c). While RS-67333 did not enhance STM under healthy conditions (saline vs. RS-67333, *p* = 1; Bonferroni’s post hoc test), it improved STM performance post-sPCP in several animals, though it did not fully restore it (RS-67333 health vs. sPCP, *p* = 0.056). Notably, some mice exhibited negative DIs, indicating a preference for familiar objects, potentially reflecting emotional distress toward novel stimuli.

Stronger effects were observed in the LTM test, where repeated-measures ANOVA indicated that memory disruption by saline after sPCP was fully rescued by RS-67333 (*n* = 7 mice; F_1,6_ = 22.052, *p* < 0.001) (Figure 4d). Similar to STM, the 5-HT4R agonist did not enhance LTM under healthy conditions (saline vs. RS-67333, *p* = 1) but completely restored LTM after sPCP (RS-67333 health vs. sPCP, *p* = 1; saline vs. RS-67333 after sPCP, *p* < 0.001; see individual DIs in Supplementary Materials).

To further investigate any effects of RS-67333 on sPCP-produced anxiety, we examined the time animals spent visiting the two objects in the maze during the STM and LTM tests. Overall, the mice visited the items more frequently during the 24 h test than the 3 min test under both healthy and sPCP conditions (34 ± 4 s vs. 23 ± 2 s, p = 0.018; unpaired *t*-test, 24 h vs. 3 min with health and sPCP conditions combined). The exploration time was the shortest after saline injection under sPCP conditions (mean 18 ± 2 s; *p* = 0.029; health vs. sPCP conditions in STM test), likely reflecting fear of exploring a well illuminated maze that contained unfamiliar items. Notably, sPCP-treated mice visited the objects more frequently after injection of RS-67333 under the same behavioral conditions (27 ± 3 s, *p* = 0.043).

Together, these findings suggest that RS-67333 reduced sPCP-induced memory impairments and anxiety, which impacted LTM more than STM.

### 2.3. 5-HT4R Partial Agonist RS-67333 Mitigates sPCP-Induced Aberrant Delta Oscillations and Coupling in the Dorsal Hippocampus and Prelimbic Cortex

In the preceding behavioral experiments, we recorded neural activity from the CA1 region of the dHPC and the PL mPFC during the hour prior to object familiarization. RS-67333 (1 mg/kg, i.p.) or saline was administered 15 min after a baseline epoch, and recordings continued for 45 min until the start of the familiarization phase (Figure 4b). We analyzed changes in neural oscillations and local synchrony in the CA1 and PL regions produced by RS-67333 relative to saline controls performed a week prior, both under healthy conditions and following sPCP treatment. Specifically, we calculated power spectral densities for delta (2–5 Hz), theta (8–12 Hz), beta (18–25 Hz), low-gamma (30–48 Hz), high-gamma (52–100 Hz), and higher frequencies (100–200 Hz). We also quantified theta–high gamma coupling in CA1, an intrinsic synchronization between the phase of theta oscillations and the amplitude of gamma oscillations (phase: 5–10 Hz, amplitude: 50–100 Hz), which is consistently observed in awake, normally behaving mice under healthy conditions [37,38]. Additionally, we assessed delta–high gamma and delta–high-frequency coupling in the PL cortex (phase: 2–6 Hz and 4–10 Hz, amplitude: 75–100 Hz and 150–200 Hz, respectively). These cross-frequency couplings typically emerge due to stress and under pathological conditions, such as acute PCP exposure [39].

**Figure 5:**
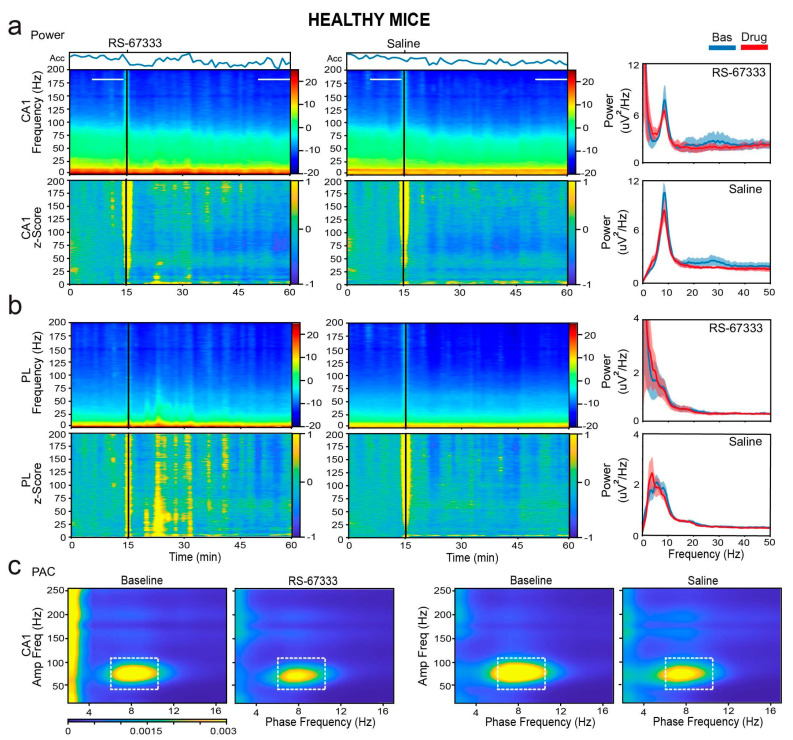
RS-67333 does not alter local synchronization in CA1 and PL in healthy mice. (**a**) Power spectrograms (power per minute) and corresponding z-scores (lower panels) of signals in the CA1 after the administration of RS-67333 (1 mg/kg, i.p.) and saline. Quantification of the animals’ mobility (variance of the accelerometer, Acc) per minute is also shown. Power spectra of signals during the last 10 min of baseline are depicted in blue, and signals from mins 35 to 45 after RS-67333 administration (50–60 min after the start of the recordings) are illustrated in red. (**b**) Same analyses for PL signals. Power at middle frequencies (theta, beta, and low and high gamma) decreased equivalently in both regions following RS-67333 and saline administrations. (**c**) Comodulation maps quantifying local cross-frequency coupling in CA1 for the 10 min epochs selected above. Dashed squares mark theta–high gamma coupling. CA1 theta–gamma coupling decreased equivalently following RS-67333 and saline administrations.

We first examined whether RS-67333 influenced the general locomotion of the mice via accelerometers integrated within the electrophysiological system (see Section 4). Since locomotion modulates neural activity, it could potentially affect neural oscillations. Under healthy conditions, both RS-67333 and saline injections resulted in a similar reduction in general activity over time (F_1,7_ = 8.344, *p* = 0.023, no interaction; repeated measures ANOVA; *n* = 6 mice). This reduction in activity was accompanied by modest decreases in power across middle-frequency bands (theta, beta, low and high gamma), particularly in CA1 (Figure 5a,b). A significant reduction in theta–gamma coupling in CA1 was also observed (F_1,7_ = 11.824, *p* = 0.011) (Figure 5c). These decreases were similar in the RS-67333 and saline groups.

Following sPCP treatment, the behavioral responses of mice to RS-67333 and saline injections differed. Animals treated with saline exhibited sustained increased activity, possibly reflecting the agitation produced by the stress of drug injection under the influence of sPCP. In contrast, RS-67333-treated animals showed a decrease in activity akin to healthy conditions (F_1,9_ = 4.857, *p* = 0.05). This suggests that RS-67333 produced anxiolytic effects, which likely facilitated better memory acquisition during the familiarization phase. In agreement with these results, sPCP-treated mice visited the objects in the maze more frequently after injection with RS-67333 than saline (see above).

**Figure 6:**
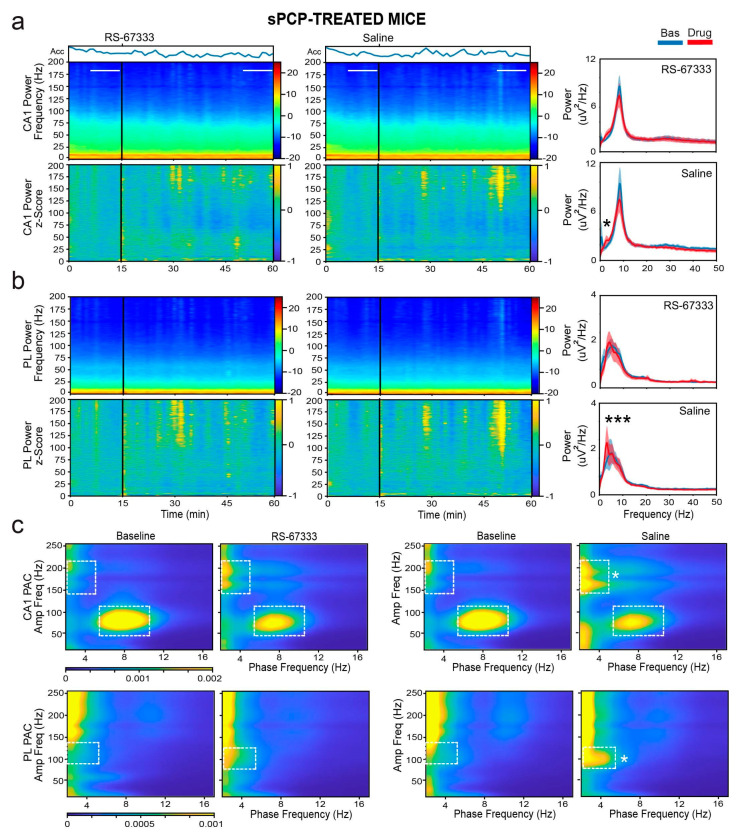
RS-67333 mitigates sPCP-induced aberrant delta synchronization in CA1 and PL. (**a**) Power spectrograms (power per minute) and corresponding z-scores (lower panels) of signals in the CA1 after the administration of RS-67333 (1 mg/kg, i.p.) and saline. Lines depict the epochs selected for signal quantification. The animals’ mobility (Acc per minute) is also shown. Right panels: power spectra of signals during the last 10 min of baseline are depicted in blue, and signals from min 35 to 45 after RS-67333 administration (50–60 min since the start of the recordings) are illustrated in red. Delta power increased in CA1 more after saline than RS-67333. Repeated-measures ANOVA followed by Bonferroni’s post hoc test (RS-67333 vs. saline, * *p* = 0.03). (**b**) Same analyses for PL signals. Delta power increased in PL after the injection of saline but not RS-67333 (RS-67333 vs. saline, *** *p* < 0.001). (**c**) Comodulation maps quantifying local cross-frequency coupling in CA1 and PL for the 10 min epochs selected above. Dashed squares mark intrinsic theta–gamma coupling in CA1 and sPCP-associated delta–high frequency coupling in CA1 and delta–high gamma coupling in PL (RS-67333 vs. saline, delta–high frequency and delta–high gamma coupling, * *p* = 0.02 and 0.045, respectively).

The differential neural oscillation changes reflected these behavioral differences. Specifically, saline injections caused significant increases in delta power in both the CA1 and PL regions, whereas the increases were substantially smaller after RS-67333, particularly in the PL cortex (F_1,9_ = 93.667, 40.602, *p* < 0.001, CA1 and PL, respectively; *p* < 0.001, saline vs. RS-67333, post hoc test with Bonferroni correction). Similar reductions in theta power were observed in both regions, particularly within the sub-band associated with locomotion (8–10 Hz; F_1,9_ = 65.373, 75.984, *p* < 0.001, CA1 and PL, respectively; *p* < 0.001, saline vs. RS-67333). Additionally, large decreases in low-gamma power were observed after saline injections, which were effectively prevented by RS-67333, especially in CA1 (F_1,9_ = 77.631, 214.496, *p* < 0.001, CA1 and PL, respectively; *p* < 0.001, saline vs. RS-67333). Beta, high-gamma, and higher-frequency bands also showed reductions, but the changes were similar between the saline and RS-67333 groups (Figure 6a,b).

Next, we examined phase–amplitude coupling changes. Consistent with the decreases in theta and gamma power, theta–gamma coupling in CA1 was reduced following both saline and RS-67333 injections, with no significant difference between the groups (F_1,9_ = 5.35, *p* = 0.046, no interaction). In contrast, sPCP-mediated increases in delta power in both regions resulted in abnormal coupling. In CA1, aberrant delta–high frequency coupling (phase: 2–5 Hz, amplitude: 150–200 Hz) emerged, whereas in the mPFC, abnormal delta–high gamma coupling (phase: 2–5 Hz, amplitude: 75–120 Hz) emerged after saline administration, likely due to the stress produced by the injection. These increases in cross-frequency synchronization were significantly lower when mice received RS-67333 (F_1,9_ = 11.479 and 9.767, *p* = 0.008, 0.005, respectively; Figure 6c).

Collectively, these results indicate that 5-HT4Rs play a role in attenuating sPCP-induced reductions in hippocampal theta and low-gamma power, as well as in restraining aberrant delta and delta–high gamma synchronization in the PL cortex. These effects were not observed under healthy conditions, suggesting that RS-67333 may rescue disrupted neural dynamics caused by sPCP. The subsequent section explores neural communication between the CA1 and PL regions in response to RS-67333 administration.

### 2.4. 5-HT4R Partial Agonist RS-67333 Does Not Influence Signal Directionality Between the Dorsal Hippocampus and the Prelimbic Cortex

**Figure 7:**
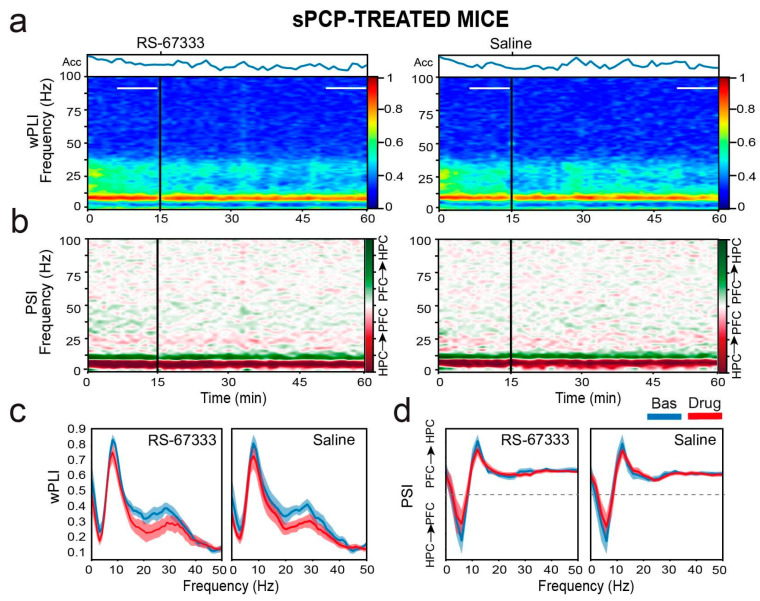
RS-67333 does not affect hippocampal–prefrontal communication in sPCP-treated mice. (**a**) Time course of changes in wPLI (phase coherence) after the administration of RS-67333 (1 mg/kg, i.p.) and saline. dHPC-mPFC theta (8–12 Hz) and low-gamma (20–40 Hz) coherence bands can be observed as red (high coherence) and light-blue (moderate coherence) signals, respectively. Phase coherence was equivalent after the injection of RS-67333 and saline. (**b**) Time course of changes in PSI (circuit directionality). The intrinsic dHPC → mPFC theta and beta bands can be observed as dark- and light-red signals, respectively. Signal directionality was equivalent after the injection of RS-67333 and saline. (**c**) Quantification of wPLI for the two experimental groups for the 10 min epochs selected above. (**d**) Corresponding quantification of PSI. Dashed lines denote zero PSI [26]. Similar results were obtained under healthy conditions.

We next examined whether RS-67333 influenced neural communication between the CA1 and PL regions before object familiarization, which could further explain its memory-restoring effects. To assess circuit synchronization, we calculated phase coherence, a measure of oscillatory alignment within the same frequency band across regions. The weighted phase-lag index (wPLI) method was applied to minimize contamination from common-source, zero-lag effects that could simultaneously impact multiple regions [28,39]. In both healthy and sPCP-treated mice, phase coherence in the theta (8–12 Hz) and low-gamma (20–40 Hz) bands was consistently detected [28]. Low gamma coherence significantly decreased after both saline and RS-67333 treatments in similar ways under healthy and sPCP conditions (Figure 7a,c).

To assess the directionality of neural signals within the circuit, we applied the phase slope index (PSI) method [28,40], which estimates the consistency (i.e., low variance) of the phase lag between two signals within a given frequency band. A stable phase relationship is indicated by lower variance compared to surrogate data, while the sign of the phase lag determines the directionality of information flow. Here, positive slopes represent dHPC → mPFC signaling (dark red), whereas negative slopes indicate mPFC → dHPC flow (green). In healthy mice, a robust dHPC → mPFC theta-band (4–10 Hz) flow is consistently observed, appearing as a broad, dark-red band in the directionality plots. In both healthy and sPCP-treated mice, pronounced dHPC → mPFC theta signaling was evident, which was unaltered by RS-67333 or saline (Figure 7b,d).

These findings indicate that sPCP did not disrupt baseline hippocampal–prefrontal network dynamics before object familiarization, nor did RS-67333 alter this balance.

## 3. Discussion

We investigated the neural substrates underlying the pro-cognitive effects of 5-HT4R activation in hippocampal–prefrontal pathways at both anatomical and functional levels. Our findings reveal that 5-HT4Rs are expressed by both PV+ and SST+ GABAergic interneurons and that their pharmacological activation attenuates aberrant neural synchronization associated with memory deficits and anxiety.

We report that 5-HT4Rs are not only expressed by pyramidal neurons in the HPC and mPFC but also by 30 to 60% of PV interneurons and 15% of hippocampal SST interneurons. Behaviorally, 5-HT4R partial agonist RS-67333 rescued long-term recognition memory deficits in the sPCP mouse model of schizophrenia but had no impact on healthy mice. At the neurophysiological level, RS-67333 attenuated sPCP-induced increases in delta oscillations and associated cross-frequency coupling in both the CA1 area of the HPC and the PL mPFC without affecting the circuit’s connectivity.

Our findings expand the expression map of the 5-HT4R protein in the mouse brain [34]. The immunohistochemical protocol was refined to detect 5-HT4R staining in brain regions with low expression. Not only did we detect high expression levels in the caudate–putamen and mossy fibers, as previously described [10,16,34], but we were able to track individual 5-HT4R+, PV+, and SST+ fibers with high signal-to-noise ratios. We report that, in addition to pyramidal neurons, 30% to 60% of PV+ interneurons co-express 5-HT4Rs in hippocampal CA1, CA3, and DG regions, whereas in prefrontal ACC, PL, and IL regions, it was between 30% and 40%. Co-labeling in SST+ interneurons was much lower, at around 15% in the HPC and less than 10% in the mPFC. We note that 5-HT4R staining in individual SST+ cells of the mPFC was at the limit of our detection levels. Therefore, it is plausible that expression of 5-HT4Rs in SST+ neurons was underestimated in both the HPC and the mPFC. Furthermore, considering the extensive arborization of individual SST+ cells in these areas, with broad staining in cortical layer 1, a role for 5-HT4Rs in this population may not be negligible. Future studies should clarify how low levels of receptor expression impact signaling in individual neurons and how this affects neural network activities. We also confirmed distinct receptor lamination in the mouse cingulate and prelimbic cortices, with denser expression in layers 2/3, as reported in the human frontal cortex [17]. Our findings are in contrast with results from the rat brain, where 5-HT4R mRNA expression was reported in hippocampal and cortical glutamatergic neurons but not in GABAergic interneurons [15,18,34].

RS-67333 was administered acutely before the familiarization phase of the NOR task to strengthen the acquisition of new memories, as previously reported in healthy animals [12] and in the sPCP model of schizophrenia for other pro-cognitive compounds [33,35]. This strategy successfully rescued memory performance in sPCP-treated mice but had no effect under healthy conditions. Moreover, while the rescue of STM by RS-67333 was variable, LTM was consistently restored across treated animals. We have previously shown impaired LTM for several weeks following sPCP treatment [26,28]; thus, RS-67333-mediated memory improvement cannot be merely explained by its testing a week after saline. Notably, higher doses of RS-67333 (5.0 and 10.0 mg/kg) facilitate both STM and LTM in healthy rats [1,12], suggesting that 5-HT4R agonism may have dose-dependent effects on memory enhancement and restoration, as well as on STM. In line with this, chronic RS-67333 administration has been shown to improve recognition memory in mice [9], indicating that prolonged 5-HT4R activation may enhance normal memory function.

Importantly, 5-HT4R activation exerted calming effects during the epoch preceding the familiarization phase, suggesting that emotional mechanisms may have also contributed to the actions of RS-67333. However, the fact that RS-67333-mediated pro-cognitive effects were more consistently observed 24 h after its administration suggests an effect of 5-HT4Rs on the molecular mechanisms underlying memory acquisition and consolidation and not merely an anxiolytic effect. We conclude that a combination of emotional and cognitive mechanisms may contribute to 5-HT4R-mediated memory-rescuing actions. These findings align with preclinical studies showing that 5-HT4R activation can alleviate cognitive deficits and exert rapid anxiolytic effects in rodent models of major depression, Alzheimer’s disease, and Parkinson’s disease [10,12,14,41,42]. Together, these results highlight the therapeutic potential of 5-HT4R activation in mitigating both cognitive and negative symptoms associated with schizophrenia.

Consistent with its behavioral effects, 5-HT4R activation attenuated sPCP-induced disruptions in hippocampal–prefrontal neural dynamics without affecting healthy animals. Specifically, RS-67333 reduced abnormal delta oscillations in CA1 and PL and attenuated delta–high-frequency and delta–high gamma coupling, respectively. These findings align with previous reports indicating that sPCP shifts hippocampal–prefrontal networks from intrinsic theta domains to pathological delta regimes [26,28,39], supporting the hypothesis that large-scale delta connectivity contributes to cognitive dysfunction in schizophrenia [43,44].

5-HT4Rs are well known to modulate functional synaptic plasticity essential for learning and memory in the HPC and PFC, including roles in long-term potentiation (LTP), long-term depression (LTD), and excitatory-inhibitory balance regulation [10,14,41,42]. Thus, 5-HT4R activation was expected to robustly influence neural oscillations and circuit synchrony relevant to memory. However, its overall effects on local oscillations, cross-frequency coupling, and hippocampal–prefrontal circuit communication were modest—smaller than those reported for other serotonin receptors such as 5-HT1A and 5-HT2A [27,39]. This was unexpected, given the consistent LTM improvements observed with RS-67333 in this study. Possible explanations include the relatively lower expression of 5-HT4Rs within this circuit and the low dose of the agonist used. Notably, 5-HT4R activation enhances acetylcholine release and increases cAMP and BDNF signaling [10], all of which likely contributed to the observed memory improvements. It is also possible that 5-HT4R-mediated memory enhancement depends on rapid changes in neural dynamics that our current analytical methods could not detect. Future studies using alternative approaches are needed to explore this further.

Our study offers new insights into the anatomical distribution and functional significance of 5-HT4Rs within the hippocampal–prefrontal pathways of mice, addressing key gaps in the field.

## 4. Materials and Methods

### 4.1. Animals

Adult female and male C57Bl6/J mice (2–3 months old, 20–30 g) were obtained from Charles River. Mice were housed under controlled environmental conditions, including a temperature of 22 ± 2 °C, relative humidity of 60%, and a 12:12 light–dark cycle (lights off from 7:30 p.m. to 7:30 a.m.). Food (standard pellet diet) and water were available ad libitum throughout the study, except during the brief behavioral and recording sessions, when the animals did not have access to food or water.

### 4.2. Immunohistochemistry

Mice were perfused with 4% PFA, and their brains were post-fixed in 4% PFA for 24 h at 4 °C. Then, the brains were cryoprotected by immersing them in 30% sucrose in PBS overnight at 4 °C until they sank. The brains were then frozen at −80 °C until they were processed. Brains were later sectioned at a thickness of 30 μm using a cryostat. Brain sections containing the dHPC and the mPFC (at least three sections per area) were washed in PBS at RT (3 times for 10 min) and later incubated with blocking solution (5% Donkey serum, 0.3% Triton X-100 and 100 mM of glycine in PBS) [45] overnight at RT. After blocking, the tissue sections were incubated in blocking solution containing the primary antibodies at 4 °C over the weekend, plus an extra hour at RT. The primary antibodies were rabbit anti-5-HT4R (1:200, BS2127R; BIOSS, Woburn, MA, USA), sheep anti-PV (1:500, PA5-47693; Invitrogen, Waltham, MA, USA), and mouse anti-SST (1:500, GTX71935; GeneTex, Irvine, CA, USA). In the case of 5-HT4R and SST assays, the sections were incubated for 60 min at RT with a solution of ReadyProbes™ Mouse on Mouse IgG Blocking Solution (1:30) Invitrogen R37621) and PBS and washed in PBS at RT (3 times for 10 min) prior to incubation with the primary antibodies to avoid nonspecific binding of antibodies to the tissue. Later, tissue sections were washed in PBS at RT (3 times for 10 min) and incubated with the secondary antibodies for 2 h at RT in the dark. The secondary antibodies were Goat anti-rabbit IgG Alexa Fluor^®^ 488 (1:1000, AB-150077; Abcam, Cambridge, UK), Donkey anti sheep Alexa Fluor™ 594 (1:1000, Invitrogen A-11016), and Goat anti mouse Alexa Fluor™ 594 (1:500, Invitrogen A-11005). Finally, the slices were cleaned in PBS (3 times for 10 min) and mounted with Fluoromount-G™ with DAPI (Invitrogen 00-4959-52) for nuclear staining, then covered with a coverslip. The sections were examined at 10× and 20× under a Dragonfly 200 confocal microscope system (Oxford Instruments Andor, Abingdon, UK) using Fusion software (version 2.4.0.14; Oxford Instruments) and at 63× with a Stellaris 8 DRIVE confocal microscope system (Leica Microsystems, Wetzlar, Germany) using Leica Application Suite X software (version 4.6.1.27508; Leica Microsystems). Negative control experiments with no primary antibodies were conducted to confirm the specificity of the stainings. The images were visualized and analyzed using ImageJ software (version 2.14.0/1.54f). The histological counts provided in Table 1 represent the average counts across multiple sections.

### 4.3. Surgical Procedures

Mice were anesthetized with 4% isoflurane and placed in a stereotaxic apparatus. Anesthesia was maintained between 0.5 and 2% throughout the procedure. Small craniotomies were drilled above the hippocampus (HPC) and medial prefrontal cortex (mPFC). Several micro-screws were inserted into the skull to stabilize the implant, with one positioned over the cerebellum serving as the general ground. Stereotrodes, composed of two twisted strands of 25 μm tungsten wire (Advent Research Materials, Witney, UK) insulated with heat-shrink tubing, were implanted unilaterally in the prelimbic (PL) region of the mPFC (AP: 1.5, 2.1 mm; ML: ±0.6, 0.25 mm; DV: −1.7 mm from bregma) and the CA1 area of the dHPC (AP: −1.8 mm; ML: −1.3 mm; DV: −1.15 mm). Neural activity was monitored during electrode placement to confirm accurate targeting of the CA1 region. Additionally, three reference electrodes were implanted in the corpus callosum and lateral ventricles (AP: 1, 0.2, −1; ML: 1, 0.8, 1.7; DV: −1.25, −1.4, −1.5, respectively). At the time of implantation, electrode impedances ranged from 100 to 400 kΩ. Electrodes were secured with dental cement and connected to an adaptor for integration with the recording system. Postoperatively, mice were given at least one week to recover, during which time they were closely monitored and received analgesia and anti-inflammatory treatments. Before experiments commenced, animals were handled and habituated to the recording cable. Following the completion of experiments, electrode placements were histologically verified using Cresyl violet staining, and data from misplaced electrodes were excluded from analysis.

### 4.4. Novel Object Recognition Test (NOR)

Recognition memory was assessed using a custom-designed T-maze, as previously reported [24,26,28]. The maze was constructed from aluminum with wider and higher arms than standard mazes (8 cm wide × 30 cm long × 20 cm high) and was shielded and grounded to accommodate electrophysiological recordings. It was positioned on an aluminum platform to minimize noise interference. Novel–familiar object pairs were validated as described in [46], and the arm in which the novel object was placed was randomized across trials. The task consisted of a habituation phase, familiarization phase, short-term memory test (STM), and long-term memory test (LTM), each lasting ten minutes (Figure 4b). During habituation, mice explored the maze without objects. After a one-hour interval, mice underwent the familiarization phase, where two identical objects were placed at the ends of the lateral arms. STM and LTM tests were conducted 3 min and 24 h later, respectively, with one familiar object and one novel object placed in the maze. Typically, initial visits and interactions with novel objects were longer compared to later visits and interactions with familiar objects [26].

### 4.5. Pharmacology

RS-67333, a potent and highly selective partial 5-HT4R agonist, was obtained from Tocris Bioscience© (Minneapolis, MN, USA) and dissolved in saline solution (0.9% NaCl). It was administered intraperitoneally (i.p.) at a dose of 1 mg/kg. Phencyclidine (PCP), a non-competitive NMDAR (N-Methyl-D-aspartate receptor) antagonist, was purchased from Sigma-Aldrich and Merck (St. Louis, MO, USA). It was administered subcutaneously (s.c.) at a dose of 10 mg/kg for 10 days (Monday to Friday over two consecutive weeks), as previously described [26].

### 4.6. Neurophysiological Recordings and Data Analyses

Electrophysiological recordings were conducted between 9:00 a.m. and 5:00 p.m. during the light cycle of housing in freely moving mice exploring their home cages (369 × 165 × 132 mm). Animals did not have access to food or water during the recording sessions but had food and water available just before and after each experiment. All the recordings were carried out with the Open Ephys system (software version 0.4.3.3) at 0.1–6000 Hz and a sampling rate of 30 kHz with Intan RHD2132 amplifiers equipped with an accelerometer. The home cage was moved from the housing room to the experimental room within the animal facility where recordings were implemented. One or two animals were recorded simultaneously in separate cages and electrophysiological setups in the same room.

Recorded signals from each electrode were detrended, notch-filtered to remove power-line artifacts (50, 100, 150 and 200 Hz), and decimated to 1 kHz offline to obtain local field potentials (LFPs). Noisy electrodes detected by visual inspection from individual channel spectrograms were not used. Power spectral density results were calculated using the multi-taper method from the spectral_connectivity package in Python (version 3.11.5; time–half-bandwidth product = 5, 9 tapers, and 60 s sliding time window without overlap). Spectrograms were constructed using consecutive Fourier transforms (scipy.signal.spectrogram function, 60 s time window, no overlap, no detrend). A 1/f normalization was applied to power spectral density results, and power spectrograms were scaled to decibels for visualization purposes. The frequency bands considered for the band-specific analyses included delta (2–5 Hz), theta (8–12 Hz), beta (18–25 Hz), low gamma (30–48 Hz), high gamma (52–100 Hz), and high frequencies (100–200 Hz, 100–150 Hz, and 150–200 Hz).

Phase–amplitude coupling (PAC) was measured with a Python implementation of the method described by Tort et al. (phase frequencies = [0, 15] with 1 Hz step and 4 Hz bandwidth, amplitude frequencies = [10, 250] with 5 Hz step and 10 Hz bandwidth) [47]. The length of the sliding window was 300 s for the overview plots and 60 s for the quantifications, without overlap. PAC quantification results were obtained by averaging the values of selected areas of interest in the comodulograms. These areas were: theta–high-gamma comodulation in CA1 (phase: 5–10 Hz, amplitude: 50–100 Hz), delta–high gamma in PL (phase: 2–6 Hz, amplitude: 75–100 Hz), and theta–high frequency in PL (phase: 4–10 Hz; amplitude: 150–200 Hz).

Prefrontal–hippocampal phase coherence was estimated via the weighted phase-lag index (wPLI, Butterworth filter of order 3), a measure of phase synchronization between areas aimed at removing the contribution of common-source zero-lag effects that allowed us to estimate the synchronization between the PFC and the HPC, mitigating source signals affecting multiple regions simultaneously [27,48,49,50]. PLI spectra were built by applying the previous function multiple times with a 1 Hz sliding frequency window (using Butterworth bandpass filters of order 3), and PLI spectrograms were generated by applying the PLI spectra function over a 60 s sliding window (without overlap). In addition, we calculated the flow of information between areas with the phase slope index (PSI) with a Python translation of MATLAB’s data2psi.m (epleng = 60 s, segleng = 1 s) from [40]. PSI spectrograms and spectra were constructed with the same strategy as PLI plots but using a 2 Hz sliding frequency window. All the results (LFP power, local and circuit PAC, and PFC-HPC wPLI) are provided as z-scores with respect to baseline statistics (i.e., data are demeaned by the baseline mean, then normalized by the baseline standard deviation).

We used the accelerometer’s signals to evaluate the effects of the drugs on the general mobility of mice. The variance of the acceleration module (Acc) quantifies the variation of movement across the three spatial dimensions. More specifically, we calculated the instantaneous module of raw x, y, and z signals from which we measured the variance of 1 min bins [24]. The Acc results are presented as a ratio to the highest value in the baseline condition of the respective experiment.

### 4.7. Statistical Analyses

All statistical analyses were performed using JASP open-source software (version 0.19.3.0). To assess memory performance under healthy conditions, we used Student’s *t*-tests to compare discrimination indices (DIs) between saline and RS-67333 treatments. To evaluate changes in DIs before and after subchronic phencyclidine treatment, we conducted repeated-measures ANOVAs, with treatment (saline, RS-67333, saline-sPCP, RS-67333-sPCP) as as the repeated factor. For accelerometer measures (Acc) and electrophysiological biomarkers (power, phase-amplitude coupling [PAC], weighted phase-lag index [wPLI], and phase slope index [PSI]), we applied repeated-measures ANOVAs, using baseline vs. drug (same recording session) as the repeated factor and treatment (saline vs. RS-67333, different sessions) as the between-subject factor. Data were quantified in 10 min epochs (minutes 5–15 and 50–60 of the hour prior to the familiarization phase). The Bonferroni method was applied to correct for multiple comparisons. Statistical significance was set at *p* ± 0.05.

## 5. Conclusions

- 5-HT4 receptors are not only expressed by pyramidal neurons in the HPC and mPFC but also by 30–60% of PV interneurons and 10–15% of SST interneurons.
- The 5-HT4R partial agonist RS-67333 improves memory performance and reduces anxiety in the sPCP model of schizophrenia.
- RS-67333 attenuates sPCP-induced increases in delta oscillations and associated cross-frequency coupling in both the CA1 area of the HPC and the PL mPFC without affecting the circuit’s connectivity.

## Figures and Tables

**Table 1:** Co-expression of 5-HT4Rs in PV+ and SST+ interneurons in dHPC and mPFC subregions.

Sub- Area	Sex	Number of Animals	Mean PV+ Count per Section	% Co- Expression	Number of Animals	Mean SST+ Count per Section	% Co- Expression
**CA1**	Female	5	28 ± 3	53 ± 10	2	14 ± 6	25 ± 14
	Male	4	23 ± 4	50 ± 6	3	16 ± 3	10 ± 7
	All	9	26 ± 3	52 ± 6	5	15 ± 3	16 ± 7
**CA3**	Female	5	19 ± 2	45 ± 8	2	14 ± 9	21 ± 15
	Male	4	22 ± 3	40 ± 5	3	7 ± 2	16 ± 11
	All	9	20 ± 2	43 ± 5	5	10 ± 4	18 ± 9
**DG**	Female	5	4 ± 1	70 ± 14	2	14 ± 9	8 ± 9
	Male	4	8 ± 1	65 ± 10	3	4 ± 1	4 ± 6
	All	9	6 ± 1	68 ± 9	5	8 ± 4	6 ± 5
**ACC**	Female	4	65 ± 22	43 ± 9	3	37 ± 9	7 ± 5
	Male	2	51 ± 17	43 ± 9	3	29 ± 6	4 ± 3
	All	6	60 ± 14	43 ± 7	6	33 ± 5	6 ± 3
**PL**	Female	4	43 ± 14	34 ± 10	3	18 ± 5	10 ± 7
	Male	2	37 ± 9	29 ± 4	3	17 ± 6	4 ± 4
	All	6	41 ± 8	32 ± 6	6	18 ± 4	7 ± 4
**IL**	Female	4	37 ± 11	43 ± 14	3	18 ± 7	18 ± 10
	Male	2	46 ± 12	42 ± 8	3	17 ± 3	6 ± 3
	All	6	40 ± 8	43 ± 8	6	17 ± 4	12 ± 6

## Data Availability

The datasets generated during and/or analyzed during the current study are available from the corresponding author upon reasonable request.

## Supplementary Materials

The following supporting information can be downloaded at https://www.mdpi.com/article/10.3390/ijms26083659/s1.
